# Maternal and Post-Weaning High-Fat Diets Produce Distinct DNA Methylation Patterns in Hepatic Metabolic Pathways within Specific Genomic Contexts

**DOI:** 10.3390/ijms20133229

**Published:** 2019-06-30

**Authors:** Laura Moody, Huan Wang, Paul M. Jung, Hong Chen, Yuan-Xiang Pan

**Affiliations:** 1 Division of Nutritional Sciences, University of Illinois at Urbana-Champaign, Urbana, IL 61801, USA; 2 Department of Human Genetics, University of California at Los Angeles, CA 90095, USA; 3 Department of Food Science and Human Nutrition, University of Illinois at Urbana-Champaign, Urbana, IL 61801, USA; 4 Department of Food Science and Human Nutrition, Division of Nutritional Sciences, University of Illinois at Urbana-Champaign, Urbana, IL 61801, USA; 5 Department of Food Science and Human Nutrition, Division of Nutritional Sciences, Illinois Informatics Institute, University of Illinois at Urbana-Champaign, Urbana, IL 61801, USA

**Keywords:** MeDIP-seq, CpG island and shore, maternal programming, insulin signaling, phosphatidylinositol signaling

## Abstract

Calorie-dense high-fat diets (HF) are associated with detrimental health outcomes, including obesity, cardiovascular disease, and diabetes. Both pre- and post-natal HF diets have been hypothesized to negatively impact long-term metabolic health via epigenetic mechanisms. To understand how the timing of HF diet intake impacts DNA methylation and metabolism, male Sprague–Dawley rats were exposed to either maternal HF (MHF) or post-weaning HF diet (PHF). At post-natal week 12, PHF rats had similar body weights but greater hepatic lipid accumulation compared to the MHF rats. Genome-wide DNA methylation was evaluated, and analysis revealed 1744 differentially methylation regions (DMRs) between the groups with the majority of the DMR located outside of gene-coding regions. Within differentially methylated genes (DMGs), intragenic DNA methylation closer to the transcription start site was associated with lower gene expression, whereas DNA methylation further downstream was positively correlated with gene expression. The insulin and phosphatidylinositol (PI) signaling pathways were enriched with 25 DMRs that were associated with 20 DMGs, including PI3 kinase (*Pi3k*), pyruvate kinase (*Pklr*), and phosphodiesterase 3 (*Pde3*). Together, these results suggest that the timing of HF diet intake determines DNA methylation and gene expression patterns in hepatic metabolic pathways that target specific genomic contexts.

## 1. Introduction

High-fat (HF) diet intake during both the pre-natal and post-natal periods has adverse effects on metabolic health, including higher body weight, reduced insulin sensitivity, systemic inflammation, and dysregulation of hypothalamic satiety cues [1,2]. Furthermore, the metabolic consequences of HF diet consumption have been in part attributed to diet-induced epigenetic modifications. For instance, post-natal HF diet exposure in rats and mice has been associated with differential methylation of the hunger/satiety genes melanocortin 4 receptor (*Mc4r*), proopiomelanocortin (*Pomc*), and leptin (*Lep*) as well as the glycotic enzymes glucokinase (*Gck*) and pyruvate kinase (*Pklr*) [3,4,5]. Perinatal HF diet also produces methylation differences in the energy homeostasis gene, peroxisome proliferator-activated receptor α (*Ppara*) [6], the inflammatory genes, toll-like receptors 1 and 2 (*Tlr-1* and *Tlr-2*) [7], and the hepatic cell cycle inhibitor, cyclin-dependent kinase inhibitor (*Cdkn1a*) [8]. 

The perinatal and post-natal periods studied in the aforementioned publications are characterized by separate epigenetic events. De novo DNA methylation is established in the perinatal period while environmental factors during the post-natal period can interfere with the maintenance of such methylation patterns [9,10,11]. Given these differences between perinatal and post-natal epigenetic processes, it is important to understand how each exposure window impacts metabolism and gives rise to a unique methylome. As opposed to using a candidate-gene approach to examine the effects of HF diet on DNA methylation, the present study instead used more comprehensive high-throughput sequencing methodologies. Genome-wide DNA methylation can be measured through using one of the methods that differentiates the methylated cytidines from unmethylated ones: bisulfite conversion, affinity capture, or endonuclease digestion. While bisulfite sequencing provides superior coverage and resolution, it is both labor-intensive and comparatively more expensive [12,13]. Alternatively, Methylated DNA Immunoprecipitation coupled with high-throughput sequencing (MeDIP-seq) is an affinity enrichment method that uses 5mC-specific antibodies to directly quantify methylated DNA sequences. MeDIP-seq offers a more cost-effective option, and integration of Methylation-sensitive Restriction Enzyme digestion followed by sequencing (MRE-seq) compensates for the limitations of MeDIP-seq by having single-base resolution and extensive coverage at regions with low CpG densities. MRE-seq is an endonuclease-based technique in which restriction enzymes identify unmethylated regions [14]. Alone, MRE-seq is confined by the sequence-specificity of existing restriction enzymes, but integrating MRE-seq with MeDIP-seq can improve coverage and resolution of genome-wide methylation and proves to be a viable way of assessing methylation in large samples [15].

The liver changes substantially throughout the early stages of life. Not only does the organ undergo structural transformation, but the functional demands before and after birth are also very different. This is reflected in the altered metabolic profiles including insulin output and hepatic enzyme kinetics in the fetus versus adult [16,17]. Therefore, the present study was designed to combine MRE-seq and MeDIP-seq to survey the effects of HF diet exposure on genome-wide methylation to further elucidate the underlying mechanisms and pathways related to hepatic metabolic adaptation. Male rats were exposed to a HF diet during either the perinatal period (maternal HF, MHF) or at post-weaning (post-weaning HF, PHF). To understand how timing of HF diet exposure impacts the hepatic epigenome, we first compared DNA methylation patterns directly between MHF and PHF rats. Using the identified differentially methylated regions (DMRs), we then compared DNA methylation and gene expression between MHF, PHF, and lifelong control-fed animals (CON) to assess whether any HF diet intake affects DNA methylation. Finally, we further examined the metabolic pathways that were potentially differentially influenced by the HF diet at perinatal and post-natal time points. 

## 2. Results

### 2.1. Compared to Maternal HF Diet Exposure, Post-Weaning HF Does Not Alter Body Weight but Induces Greater Hepatic Lipid Accumulation 

**Figure 1:**
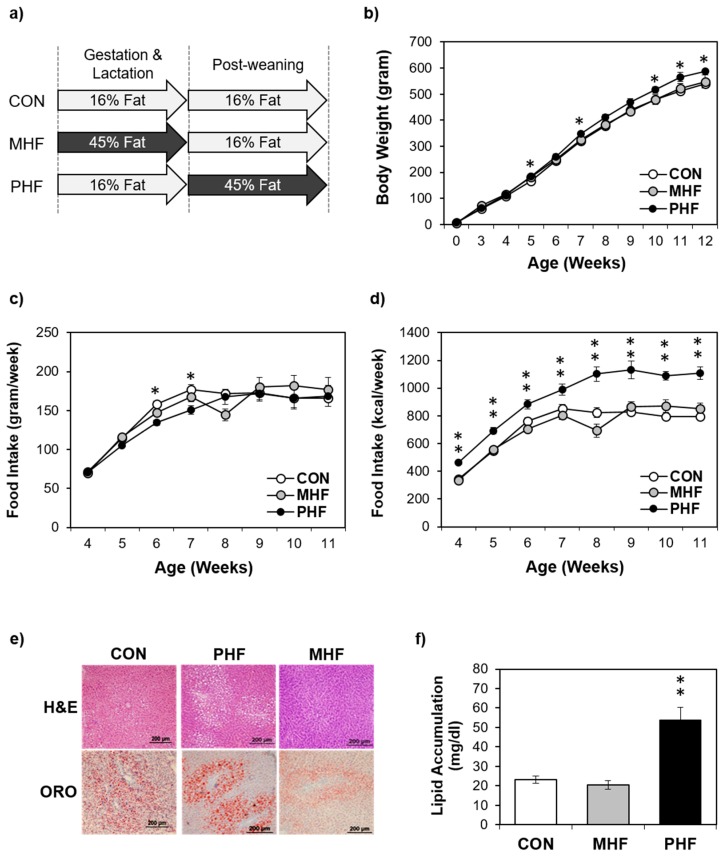
Physiological differences between perinatal and post-weaning HF diet exposure. (**a**) The experimental design involved pregnant dams that were fed either a HF diet (45% kcal from fat) or a standard AIN93G control diet throughout gestation and lactation. At weaning, male pups from control diet-fed dams were weaned onto either the same control diet or HF diet, and male pups from HF-fed dams were weaned onto the control diet, creating three groups of pups, control (CON; *n* = 8), post-weaning high fat (PHF; *n* = 9) and maternal high fat (MHF; *n* = 7), respectively. (**b**) Body weight was measured weekly during the post-weaning period. (**c**) Weekly food intake was monitored during the post-natal period. (**d**) Caloric intake as kcal/week was calculated based on the grams of food intake. (**e**) Liver cross-sections were stained with hematoxylin and eosin (H&E) (top) and Oil Red O (ORO; bottom). (**f**) PHF animals had greater hepatic lipid accumulation than MHF and CON rats. Lipid accumulation was normalized to total protein. Data points represent mean ± standard error of the mean (SEM). * denotes a significant difference (*p* < 0.05) between PHF and CON. ** denotes a significant difference (*p* < 0.01) between PHF and CON as well as between PHF and MHF.

To assess the potential differential effects of HF exposure at different developmental stages, male rats were exposed to HF diet either during gestation and lactation (maternal high fat: MHF) or after weaning (post-weaning high fat: PHF) (Figure 1a). Additionally, a control group (CON) was fed an AIN93G diet during both the perinatal and post-weaning periods. Food intake in grams per week was not significantly different between the three groups, except on week 6 and 7, when CON animals consumed more than PHF rats (Figure 1c). However, given the higher caloric density of the HF diet, the PHF group consistently consumed more calories during the post-weaning period than both CON and MHF (Figure 1d). PHF rats were only significantly heavier than CON, but not MHF, on post-natal weeks 5, 7, 10, 11 and 12 (Figure 1b). Food efficiency (weight gain/kcal intake) was lower in PHF compared to both CON and MHF (Figure S1). ORO staining revealed that lipid accumulation was unchanged in MHF compared to CON, but was significantly higher in PHF (Figure 1e,f). To account for differences in body weight and lipid accumulation, we measured expression of de novo lipogenesis and triglyceride synthesis genes and found decreased levels in PHF and increased levels in MHF (Figure S2).

### 2.2. Timing HF Diet Exposure Determines Distinct Methylation Patterns 

**Figure 2:**
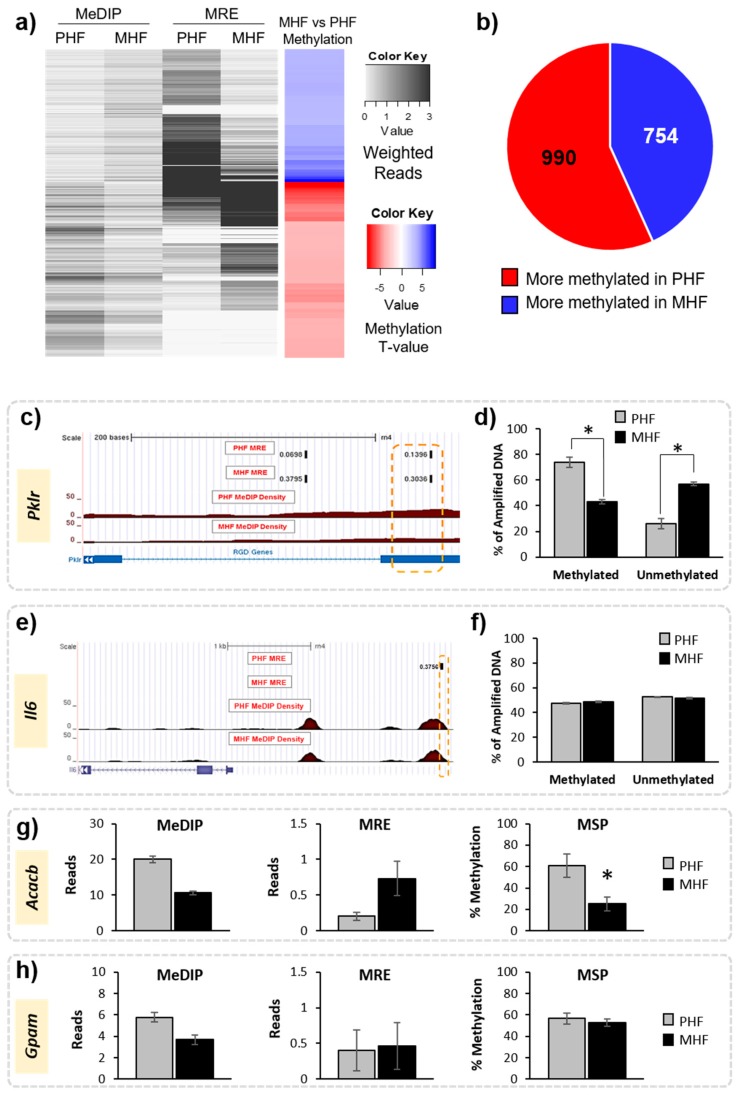
Identification of differentially methylated regions in maternal and post-weaning HF diet groups. (**a**) Heatmap shows the weighted MeDIP and MRE reads in the MHF and PHF conditions (black and white), where darker shades represent more reads. Overall methylation is shown in the far-right column. Blue represents higher methylation in the MHF group compared to the PHF group and red represents higher in the PHF group compared to the MHF group (FDR *q*-value < 0.05). (**b**) Pie chart shows overall numbers of DMRs that are more methylated in MHF animals (blue, *n* = 754) and that are more methylated in PHF animals (red, *n* = 990). (**c**–**f**) Probe-based analysis of DNA methylation was used to validate results from MeDIP and MRE-seq. (**c**) Representative tracks from the Genome Browser showing a DMR within the *Pklr* gene that was identified by MeDIP-seq and MRE-seq analysis to be more methylated in the PHF group. The region of interest used for validation is outlined in orange. The top tracks show the MRE reads in black. The red track indicates the MeDIP reads for each group. The blue track represents annotated genes and indicates the DMR position relative to exons (blue bar) and introns (blue hashed line). (**d**) A probe-based analysis of DNA methylation was used to validate the DMR. The PHF group had a higher percentage of methylated DNA (% of amplified DNA) and a lower percentage of unmethylated DNA compared to the MHF group (* *p* < 0.00001). (**e**) Representative tracks from the Genome Browser showing a genomic region upstream of the *Il6* gene identified by the joint MeDIP-seq and MRE-seq analysis as being not differentially methylated in the computational analysis. (**f**) Methylation in the *Il6* locus was measured using probe-based analysis of DNA methylation. No difference was detected between the MHF and PHF groups in this region (*p* = 0.2). (**g**) MSP was used to confirm methylation status of a region in the *Acacb* gene identified as significantly differentially methylated by MeDIP-seq and MRE-seq (*p* = 0.007). (**h**) MSP was used to validate no change in methylation status of a DMR within the *Gpam* gene that was not identified to be significant by MeDIP-seq and MRE-seq (*p* = 0.7). All bar graphs show mean ± SEM.

We first compared DNA methylation patterns directly between MHF and PHF to identify regions that differ according to timing of HF diet exposure. Genome-wide DNA methylation was measured in liver using MeDIP-seq and MRE-seq. MeDIP-seq produces extensive CpG coverage while MRE-seq offers high resolution such that combining the two methods has been proven to improve detection quality [18]. Sequencing produced over 49 million filtered MeDIP reads and over 31 million filtered MRE reads for each animal. The R-package MethylMnM was used to integrate reads from both methods to provide a comprehensive analysis of differences in methylation profile between groups. We uncovered 1744 DMRs (FDR *q*-value < 0.05), of which 990 (57%) were more methylated in the PHF group and 754 (43%) were more methylated in the MHF group (Figure 2a,b). These DMRs were distributed fairly evenly across 21 chromosomes (Data not shown). 

Methylation results were validated in multiple animals from each of the groups using both a probe-based PCR method and MSP. First, a DMR located within a gene of interest, pyruvate kinase (*Pklr*), was verified using a fluorescent probe-based PCR technique (Figure 2, Table 1). Using the fluorescent probe, we confirmed that the MeDIP- and MRE-identified DMR was highly methylated in PHF (74% methylated) when compared to MHF (43% methylated, Figure 2c,d). We used the same method to examine a genomic location upstream of the *Il6* gene that was not differentially methylated according to MeDIP and MRE analysis. Probe-based analysis of DNA methylation confirmed this result, as the methylation in the MHF (48% methylated) and PHF (47% methylated) groups were not significantly different (Figure 2). Additionally, we used MSP to confirm that a region within the *Acacb* gene was differentially methylated between PHF and MHF rats (Table 1). PHF animals had higher methylation than MHF animals (70% and 25%, respectively; Figure 2). MSP also confirmed that a region in the *Gpam* gene was not differentially methylated, as predicted by MeDIP-seq and MRE-seq (PHF: 57%, MHF: 53%). Given the strict criteria set by the MethylMnM package and the results from the subsequent validation both in this study and in previous work [19], we assume that any MeDIP- and MRE-identified DMRs represent true differential methylation between groups.

**Figure 3:**
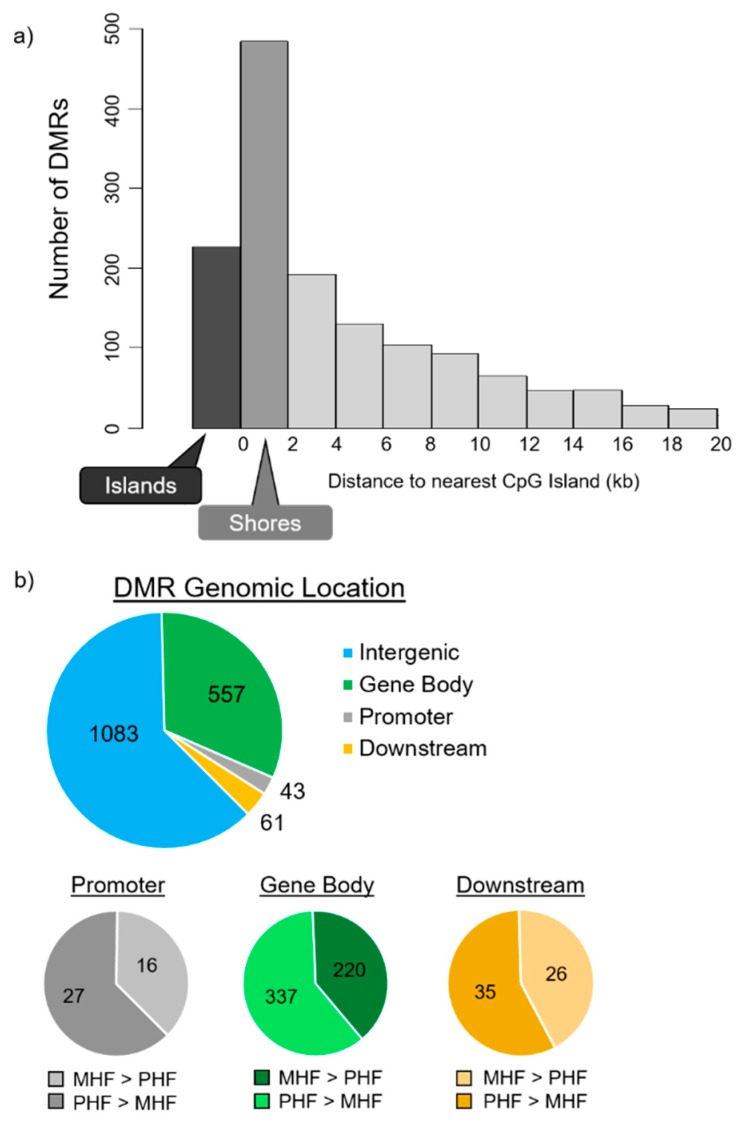
DMR distributions in relevance to CpG islands and to gene locations and structures. (**a**) DMR distribution around CpG islands. CpG shore was defined as the 2000 base pair sequence flanking either side of a CpG island. DMRs in the shores (dark gray, *n* = 484) outnumbered the DMRs located within CpG islands (black, *n* = 227). (**b**) DMR locations relative to gene structures. Promoter and downstream regions were defined as ± 1500 bp upstream of the transcription start site and downstream of the transcription end site, respectively. Top panel depicts the number of DMRs not associated with a gene (blue, *n* = 1083) and DMRs located within the gene body (red, *n* = 557), within the promoter (gray, *n* = 43), and within the downstream region (yellow, *n* = 61). A majority of DMRs are not associated with protein-coding genes. Bottom panels classify the DMRs within the promoter, gene body, and downstream regions. DMRs that are more methylated in MHF (MHF > PHF) are in darker shades, while those that more methylated in PHF (PHF > MHF) are in lighter shades.

Next, we examined DMR location relative to CpG islands. Despite the relative enrichment of DMRs in island regions, most of the differential methylation was observed in the 2000 bp shore sequences flanking the islands. In fact, more than twice as many DMRs were identified in shore regions (Figure 3a, *n* = 484, 27.8%) than in CpG islands (*n* = 227, 13.0%). This is consistent with previous reports showing that most treatment-induced changes of DNA methylation does not occur within the conserved CpG islands, but rather is localized primarily to nearby regions with slightly lower CpG densities, including the shores [20,21]. 

DMRs were then aligned with gene positions. Of the 1744 identified DMRs, 661 (38%) were associated with protein-coding genes (Figure 3b). Among the 661 gene-associated DMRs, only 43 were positioned within the 1500 bp upstream promoter region and 61 were in the 1500 bp downstream regions. The rest of the 557 gene-associated DMRs are located within intragenic regions. Furthermore, 37%, 40%, and 43% were more methylated in the MHF group than the PHF group, and 63%, 60%, and 57% were more methylated in the PHF group than the MHF group within the promoter, gene body, and downstream regions, respectively. 

A majority of the DMRs identified (*n* = 1,083, 62%) were not associated with a protein-coding gene. These regions were further examined for non-coding RNA sequences using the Genome Browser rat dataset, and only one DMR was located in an RNase P. While it is possible that these non-gene-associated DMRs are positioned within regions of “junk DNA”, it may be the case that these regions serve regulatory functions for unknown gene targets. 

DMGs were annotated with Gene Ontology terms and Kyoto Encyclopedia of Genes and Genomes (KEGG) pathways. Significant clustering was observed based on functional similarity (Figure S3). When all DMRs were considered, 8 annotation clusters were identified (Enrichment Threshold > 2.0, *p* < 0.01). Representative annotation terms with the smallest *p*-value from each cluster included ion binding (GO:0043167, Cluster 1), cell morphogenesis (GO:0000902, Cluster 2), cell fraction (GO:0000267, Cluster 3), cell morphogenesis involved in differentiation (GO:0000904, Cluster 4), stereocilium (GO:0032420, Cluster 5), regulation of synaptic transmission (GO:0050804, Cluster 6), triglyceride biosynthetic process (GO:0019432, Cluster 7), and cAMP catabolic process (GO:0006198, Cluster 8). 

Investigation of KEGG pathways demonstrated that seven pathways were DMR-enriched (Table 1, Fisher’s exact *p*-value < 0.05). Using Benjamini–Hochberg correction, only the phosphatidylinositol (PI) signaling system (rno04070) was significantly enriched (corrected *p*-value = 0.006). Other pathways of interest included insulin signaling pathway (rno04910), axon guidance (rno04360), pathways in cancer (rno05200), purine metabolism (rno00230), glycerolipid metabolism (rno00561), and Fc gamma R-mediated phagocytosis (rno04666). There were three pathways that were more methylated in the MHF group: phosphatidylinositol signaling system (rno04070), apoptosis (rno04210), and Fc gamma R-mediated phagocytosis (rno04666). On the other hand, the insulin signaling pathway (rno04910) was the only pathway that is more methylated in the PHF group.

### 2.3. Post-Weaning HF Increases Gene Expression While Maternal HF Decreases Gene Expression in the PI and Insulin Signaling Pathways 

**Figure 4:**
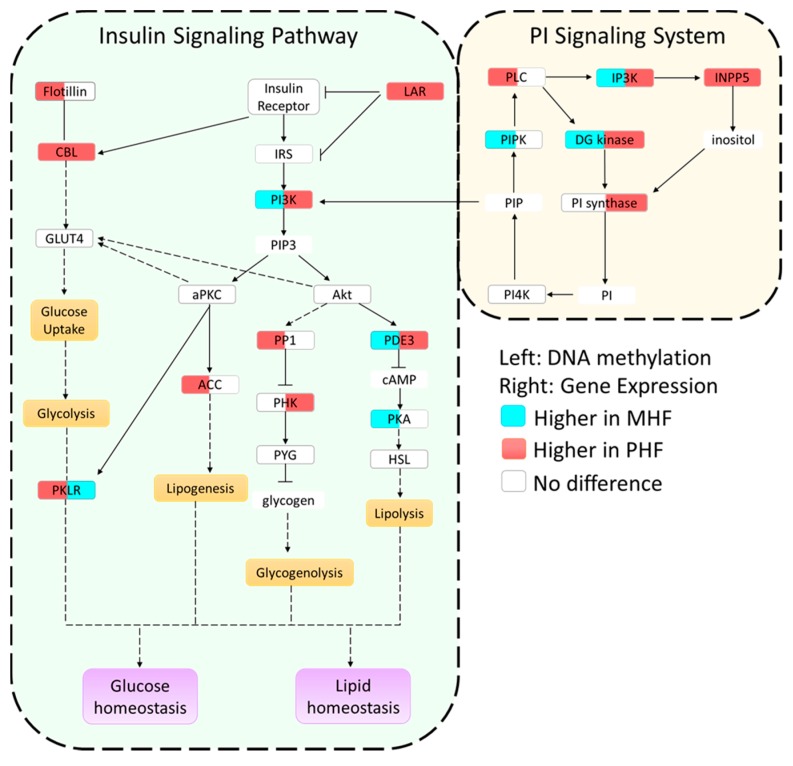
Summary of epigenetic and gene expression differences in the insulin and PI signaling pathways. Genes in each pathway are boxed and filled with two colors. The color in the left side of the box indicates methylation levels, while the color in the right side of the box denotes gene expression levels. White color signifies no change, blue color signifies higher methylation or expression in MHF animals, and red signifies higher methylation or expression in PHF animals. Yellow and purple boxes represent metabolic functions. A solid line represents a direct relationship, a dashed line represents a multi-step process, and compounds with no outline are other important signaling molecules.

From the pathway analysis, we chose to further investigate the PI signaling and insulin signaling pathways. Not only are these two pathways interconnected, but they also serve metabolic roles that are known to be perturbed by high calorie diets. Furthermore, the PI and insulin signaling pathways were statistically the most DMR-enriched according to the DAVID analysis (*p* = 0.006 and *p* = 0.4, respectively, Table 2). Additionally, we compared methylation and gene expression results to the CON group in order to assess not only differences in timing of the HF exposure, but also to examine whether any HF diet intake might disrupt metabolic pathways compared to control diet (Figure 4). 

**Figure 5:**
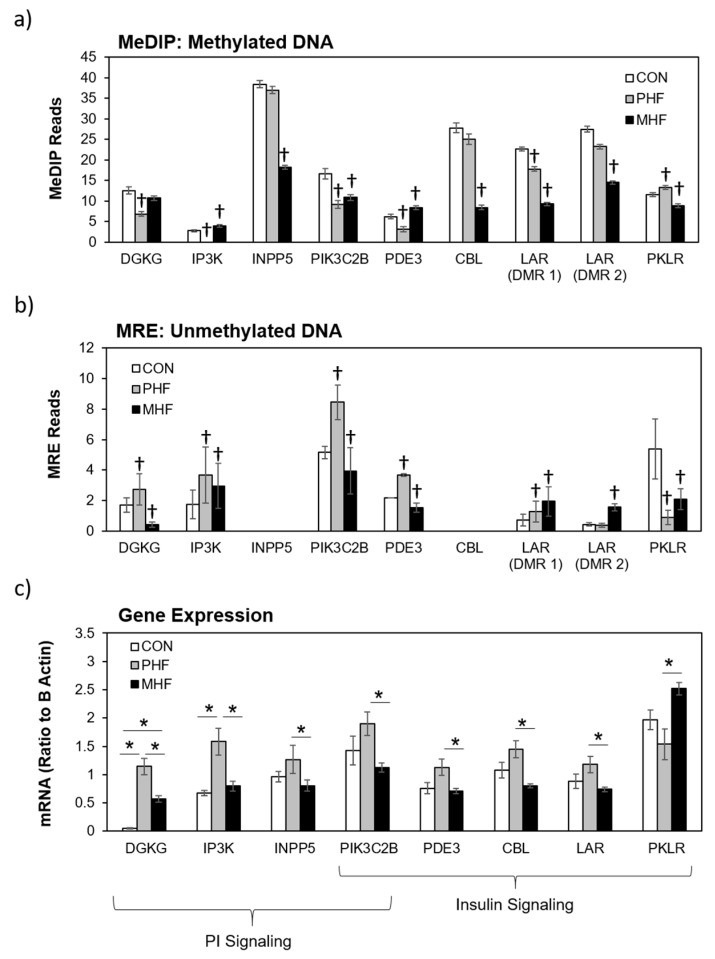
Differential gene expression in the insulin and PI signaling pathways. (**a**) Methylated DNA reads were measured using MeDIP-seq. † denotes a ≥15% difference between CON and either PHF or MHF. Data are presented as mean ±SEM across each DMR. (**b**) Unmethylated DNA was measured using MRE-seq. † denotes a ≥15% difference between CON and either PHF or MHF. Data are presented as mean ±SEM across each DMR. (**c**) Eight DMGs were significantly differentially expressed between MHF and PHF groups. The bar graph represents the mRNA quantity relative to B Actin. Data are presented as mean ±SEM. * *p* < 0.05.

We combined and condensed the pathways by focusing on 27 genes, 17 of which were differentially methylated and 10 of which were not differentially methylated but served crucial metabolic functions (Figure 4). We first examined the methylation in each of the genes relative to the CON group (Figure 5a,b). Generally, methylation of *Dgkg*, *Ip3k*, *Pik3c2b*, *Pde3*, and *Lar* was lower in PHF than CON while methylation of *Pklr* was higher in PHF than CON. Additionally, methylation was lower in *Inpp5*, *Pik3c2b*, *Cbl*, *Lar*, and *Pklr* in MHF than CON while methylation of *Ip3k* and *Pde3* was higher in MHF than in CON. We then performed quantitative PCR for all 27 genes and found 10 that were differentially expressed (*p* < 0.05). Among the differentially methylated genes, diacylglycerol kinase gamma (*Dgkg*), inositol-triphosphate 3-kinase (*Ip3k*), inositol polyphosphate-5-phosphatase (*Inpp5*), PI3 kinase (*Pi3kc2b*), phosphodiesterase 3 (*Pde3*), Cbl proto-oncogene (*Cbl*), and leukocyte antigen-related protein tyrosine phosphatase (*Lar*), were up-regulated in PHF rats, while pyruvate kinase (*Pklr*) was up-regulated in the MHF group (Figure 5c). Of the 10 genes that were not differentially methylated, phosphorylase kinase (*Phk*) and phosphatidylinositol synthase (*PI synthase*) were more highly expressed in the PHF group. From this differential gene expression, we were able to visually represent changes within glucose and lipid homeostatic processes between perinatal and post-weaning HF diet (Figure 4).

### 2.4. Genomic Context of DNA Methylation Is Indicative of Gene Expression 

**Figure 6:**
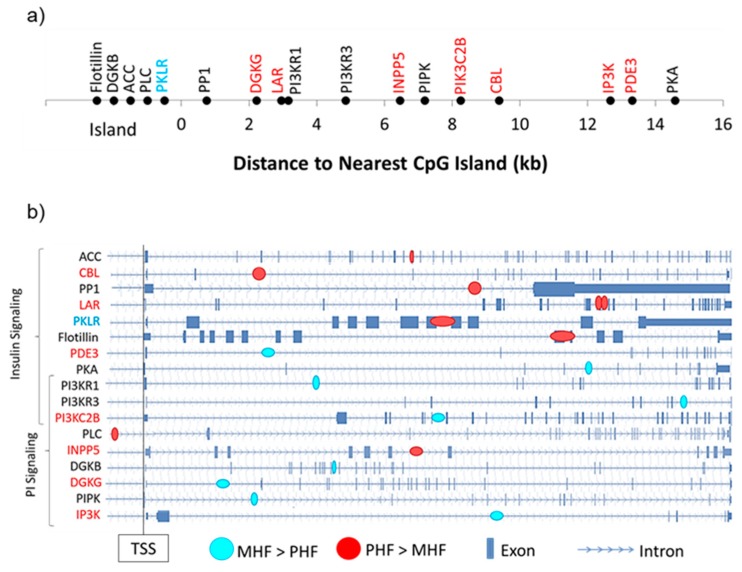
Genomic context of DMRs. (**a**) DMGs within the PI and insulin signaling pathways were mapped according to their distance from the nearest CpG island. Gene names in red and blue denote those that were more highly expressed in the PHF and MHF group, respectively. (**b**) Intragenic DMR position is shown for each DMG in the PI and insulin signaling pathways. Blue circles represent DMRs in which DNA methylation was higher in the MHF group. Red circles represent DMRs in which DNA methylation was higher in the PHF group. Gene names in red and blue denote those that were more highly expressed in the PHF and MHF group, respectively.

Among the 17 DMGs of interest, we examined the distance of the gene-associated DMR to the nearest CpG island as well as their intragenic locations (Figure 6). Of the 8 differentially expressed DMGs, only one contained a DMR that was located within a CpG island *(Pklr*, Figure 6a). Interestingly, the remaining 7 DMRs fell outside the 2000 bp shore region (Figure 6a). We also found that except for one, all DMRs were intragenic, with a majority located in introns (Figure 6b).

**Figure 7:**
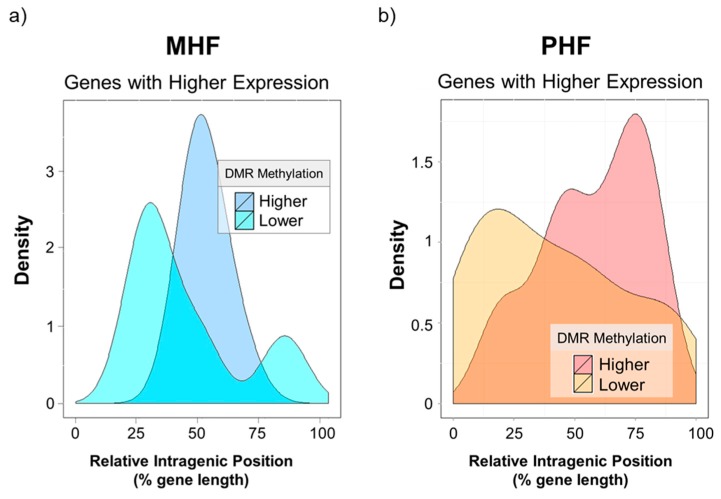
Gene expression is associated with intragenic DMR position. X-axis indicates intragenic DMR position relative to the gene’s transcription start site (x = 0) and transcription end site (x = 100). Y-axis represents the density of DMRs. (**a**) the density plot depicts DMGs that were more highly expressed in the MHF group. Higher gene expression in MHF was associated with lower DNA methylation in the middle of the intragenic region. (**b**) The density plot shows DMGs that were more highly expressed in the PHF group. Higher PHF expression was associated with higher downstream DNA methylation and lower DNA methylation near the TSS.

Lastly, we investigated the intragenic locations of these differentially methylated and differentially expressed regions. Based on qPCR results, a gene expression t-score was calculated for every gene in every animal. Values that were four standard deviations away from the mean were chosen. Relative intragenic position of a given DMR was calculated by dividing the distance of the DMR from the transcription start site (TSS) by the total length of the gene (Figure 7 x-axis). The kernel density estimation was plotted to visualize the distribution of intragenic DMR positions. Interestingly, none of the differentially expressed DMR was in the promoter regions (data not shown). Genes highly expressed in the MHF group tended to be less methylated, and this low methylation was found almost exclusively in the middle of the intragenic region (blue curves, Figure 7a). Furthermore, higher gene expression in the PHF group was correlated with lower methylation near the TSS and higher methylation in downstream regions closer to the transcription end site (TES, yellow vs red curves, Figure 7b). However, we highlight the idea that gene body methylation may play an activating or repressive role in transcriptional regulation [22].

## 3. Discussion

The present study used deep sequencing technologies to examine DNA methylation profiles in animals exposed to HF diet at different time points. First, to address whether timing of HF diet exposure induced methylation changes, we compared between MHF and PHF rats. Over 1700 DMRs were identified, of which a large portion were in CpG shores and intragenic regions. Gene-associated DMRs also clustered in the insulin and PI signaling pathways. We then compared DNA methylation in the identified regions to lifelong control-fed animals to uncover the impact of HF intake regardless of timing. In these metabolic pathways, we conclude that any HF diet decreases DNA methylation, as both the MHF and PHF groups generally had lower methylation than CON; however, expression of key metabolic regulators tended to be higher in PHF and lower in MHF. Finally, we examined DMR location relative to CpG islands and gene features. We found the greatest number of DMRs located in CpG shores and showed an inverse correlation between gene expression and distance of intragenic DNA methylation from the TSS.

The current study used two diets. The control and HF diets had equal amounts of soybean oil, but the HF diet contained higher amounts of lard. Lard provides more saturated and monounsaturated fat compared to soybean oil. This has been shown to increase adiposity and markers of inflammation [23]. Chronic obesity-induced inflammation also underlies insulin resistance [24]. Interestingly, we found differential methylation in the insulin signaling pathway, so inflammatory factors may be a potential link between HF diet and epigenetic modifications. This can be tested in future experiments using HF diets with different amounts of lard, soybean oil, or fish oil to distinguish the effects of inflammation versus obesity. All other dietary components were kept constant, except for the major carbohydrate source, which was cornstarch in the control diet and sucrose in the HF diet. Sucrose and cornstarch have been shown to differentially impact body weight gain, circulating lipid profile, insulin sensitivity, and hepatic gene expression [25,26,27]. Thus, it is possible that the observed effects could have resulted from dietary carbohydrate differences rather than fat intake, so further investigation should examine individual dietary components to better understand their effects on DNA methylation.

Overall, we observed higher body weight and hepatic lipid accumulation in PHF rats. At post-natal week 12, the PHF group weighed more than CON, but there was no difference in body weight between MHF and PHF. Given the large difference in caloric intake, this finding was surprising. However, while HF diet administration has been shown to induce weight gain during adulthood, high energy intake during the post-weaning period results in only moderate body weight differences [28,29,30,31]. During the post-weaning period, rapid growth occurs, which may mask any additional diet-induced weight gain. We found greater hepatic lipid deposition in the PHF group compared to both CON and MHF. To understand the potential mechanisms mediating these differences, we measured expression of genes that were related to de novo lipogenesis and triglyceride synthesis. We found higher expression in MHF animals and lower expression in PHF rats. The down-regulation of these pathways in PHF animals is not surprising and is likely a direct effect of abundant dietary fat and a reduced need for de novo synthesis. However, the influx of dietary fat likely leads to the increased quantity of hepatic lipid that we observed. On the other hand, MHF rats had higher expression of de novo lipogenesis genes than controls, despite the eating the same post-weaning diet. Previous reports have similarly found increased acetyl-CoA carboxylase (Acc) expression in liver and adipose tissue of maternal HF-fed animals [32,33]. Moreover, disturbances in maternal diet have been shown to drive metabolic outcomes via epigenetic programming [34,35]. In our study, it is unclear whether this was due to maternal programming, considering that there were no methylation differences at these loci at post-natal week 12. Moreover, there were no differences in hepatic lipid accumulation between MHF and CON. Thus, while gene expression might suggest that the MHF group is more efficient at converting excess energy to fat, there are other factors that prevent hepatic accumulation. Future work should quantify lipid export and size of different adipose depots to find whether changes in the liver could affect other organs.

Among 1744 DMRs identified across 21 chromosomes, ~28% of which were located within CpG shores, more than twice the number located in CpG islands (13%). This is in line with previous studies illustrating that DMRs within the same tissue type occur more frequently in shores versus islands [20,21]. We also found many DMRs in intragenic and intergenic regions as opposed to promoter regions, which is expected, as CpG islands tend to be localized in promoters [36]. Although a majority of the DMRs resided in intragenic regions, only one corresponded with an annotated non-coding RNA. We attribute this finding to the fact that information regarding the function of intergenic regions is sparse, especially for the rat. For example, the microRNA database, mirBase, annotates 4694 miRNA sequences in humans and 3232 in mouse, but only 1318 in rat. As non-coding regions become more widely studied and compiled in other model organisms, we suspect that our identified intergenic regions will show more regulatory functions. Over the past decade, ENCODE and similar projects have gradually uncovered the intricacies of the vast genetic regulatory network [37]. Most of these advances have focused on human transcriptional regulators, but as the data becomes publicly available for other model organisms we might be able to assign function to previously unnamed genomic regions.

Functional clustering and pathway analysis revealed DMR enrichment in the insulin and PI signaling pathways. Both are nutrient-sensing pathways important in carbohydrate and lipid metabolism, suggesting that maternal and post-weaning HF dietary exposure may cause distinct metabolic outcomes. Indeed, PHF animals had greater lipid accumulation in the liver than did MHF animals. In the MHF group, we also observed higher methylation and lower gene expression of *Pi3k* and *Pde3*, two genes involved in a phosphorylation cascade that inhibits lipolysis. Three separate PI3K genes were more highly methylated in the MHF compared to the PHF group, including PI3K regulatory subunit 1 (*Pi3kr1*), PI3K regulatory subunit 3 (*Pi3kr3*), and PI3K catalytic subunit type 2 beta (*Pi3kc2b*). While *Pi3kr1* and *Pi3kr3* expression was modestly decreased in the MHF group, only *Pi3kc2b* expression was significantly reduced. PI3K is a key component of the insulin signaling pathway, but its regulation is also controlled in large part by PI signaling [38]. Previous studies have shown that when members of the PI signaling system are knocked down, PI3K activity and the insulin signaling pathway are dysregulated. For example, Inositol Polyphosphate Phosphatase-Like 1 (*Inppl1*) knock out mice display insulin hypersensitivity, increased levels of phosphorylated AKT, and protection against diet-induced obesity [39,40]. Moreover, knocking down inositol polyphosphate-4-phosphatase type II (*Inpp4b*), a component of the PI signaling pathway, results in the deregulation the PI3K-AKT pathway such that the magnitude and duration of insulin-stimulated AKT activation are significantly altered [41]. In our experiment, we observed four PI signaling genes (*Ip3k*, *Inpp5*, *DG kinase*, and *PI synthase*) that were more highly expressed in the PHF than MHF group. This difference in PI signaling genes may reflect the distinct regulation of PI3K between the two groups. The observed differences in methylation of PI3K as well as the gene expression changes of its regulators in the PI signaling pathway indicate that PI3K’s function as a catalytic protein may be altered in PHF compared to MHF diet exposure. 

In the MHF group, we observed higher methylation within the first intron of *Pde3*, which was associated with the decreased expression of *Pde3*. Inhibition of *Pde3* sufficiently blocks the antilipolytic action of insulin via its role in hydrolyzing cAMP and cGMP [42]. In fact, an adipocyte Pde3 knockout has been shown to result in higher levels of insulin-stimulated lipogenesis [43]. Similarly, knockout mice had altered phosphorylation state of insulin- and cAMP-signaling components as well as higher lipolysis upon catecholamine stimulation. Thus, the decrease in *Pde3* expression in the MHF group suggests that insulin may have less of an inhibitory effect on the catabolism of triacylglercerol and contribute to the lower hepatic lipid accumulation in the MHF group. 

In addition to identifying genes that have annotated functions in PI and insulin signaling pathways, our results corroborate GWAS analyses that have investigated the association between novel loci and traits such as body–mass index (BMI), waist circumference, triglyceride levels, cholesterol and diabetes [44,45,46]. Specifically, previous studies have found that SNPs within *Cdh23* were correlated with BMI [45], SNPs in *Tph2* were correlated with obesity [44], SNPs in *Prox1* were correlated with glucose metabolism [47], SNPs in *Lipc* were correlated with cholesterol [48,49,50], and SNPs in *Pnpla3* were correlated with hepatic lipid content [51]. We found DMRs associated with each of these genes: *Cdh23*, *Tph2*, and *Lipc* were more methylated in MHF while *Prox1* and *Pnpla3* were more methylated in PHF. Although reproducibility across GWAS studies is often lacking, we provide evidence that strengthens past findings. Furthermore, we suggest that future research may benefit from combining multiple sequencing modalities to identify robust biomarkers of metabolic health.

Beyond analyzing the role of HF diet in controlling metabolic processes, we also attempted to characterize the differences in epigenetic landscape between MHF and PHF and to examine how DNA methylation is associated with gene expression. We identified that most DMRs were in CpG shores. CpG island methylation has been studied extensively in the context of islands at TSSs where higher island methylation represses gene expression. Reports have shown that intragenic CpG islands are more likely to contain alternative promoters and other regulatory features [14,52]. Differential DNA methylation in CpG shores has also been shown to be associated with gene expression [20], but it is unknown whether CpGs outside CG-dense regions perform similar regulatory functions. In our study, we also found that out of the DMRs associated with insulin and PI signaling, five were located in CpG islands, one was in a CpG shore, and the remaining 11 were more than 2 kb away from the nearest island in the open sea. CpGs in the open sea have been shown to be generally hypomethylated [53,54,55,56]. While in cancer, this hypomethylation has been associated with chromosomal instability, open sea methylation also naturally changes with during development. From birth to age 10, CpG islands tend to gain methylation whereas open sea CpGs tend to lose methylation [53,57,58,59,60,61]. Thus, it may be the case that the identified DMRs are a result of diet-mediated alterations in developmental processes. Future work should focus on defining the role of DNA methylation outside of CpG islands to understand how metabolic disturbances impact such loci.

Through the investigation of the relationship between intragenic DMR location and gene expression, we observed that all differentially expressed DMGs contained DMRs in the gene body. Five out of 8 differentially expressed DMGs contained a DMR exclusively in an intronic region while the remaining 3 genes had a DMR spanning both an intron and an exon. The role of intronic DNA methylation is not well understood. Past investigation has hypothesized that it controls alternative splicing [62,63]. Others have reported regulatory elements within introns that when methylated have either repressive or activating role in gene expression [64,65]. Additionally, reports have speculated that the balance between intronic and exonic DNA methylation to be important in determining nucleosome spacing and subsequent Pol II binding [66,67,68,69]. Thus, it is difficult to discern which mechanism is at play in each of the identified genes.

There is currently no clear consensus as to the function of gene body DNA methylation. It has been proposed to mediate chromatin structure, splicing, and transcriptional kinetics; however, it is clear that gene body methylation does not necessarily act in the same way as traditionally studied promoter methylation [70]. We observed that DMR distance from the TSS was associated with gene expression. Higher expression was associated with higher methylation in more distal regions. These findings are supported by previous reports that gene expression increased with higher gene body methylation, but did not distinguish among different intragenic locations [71,72]. Other studies showed that highly expressed genes were characterized by low methylation in the gene body near the TSS and high intragenic methylation closer to the 3’ TES [22,73]. Similarly, low gene expression is correlated with high methylation in the first exon [74].

HF diet may affect the epigenetic landscape via numerous mechanisms. HF diet can incite inflammation and hormonal changes that have been associated with altered DNA methylation patterns [75]. However, it may be the case that HF diet acts directly on epigenetic modifiers and methylation processes. For instance, short-chain fatty acids such as butyrate and acetate have been shown to inhibit histone deacetylases (HDACs) and alter DNA methylation [76]. Furthermore, acetyl-CoA, the end-product of beta oxidation, is a rate-limiting cofactor in histone acetylase (HAT) activity [77]. Due to the bidirectional recruitment and interaction between histone modifications and DNA methylation, HF diet has the potential to perturb chromatin state and severely impact transcription. 

The observed methylation discrepancies that occur during the perinatal versus post-weaning periods may be explained by the drastic differences in methylation events between the two groups. During the perinatal period, an initial wave of demethylation is followed by de novo methylation by methyltransferases DNMT3a and DNMT3b [9]. However, during the post-weaning period, methylation patterns have already been established, and further upkeep relies solely on the maintenance DNA methyltransferase DNMT1 [78]. HF diet may interfere differently with certain types of methyltransferases. Previous reports have shown that altering dietary folate and choline has different effects on the expression of DNMT1 and DNMT3 and that these changes vary with length and timing of exposure [79,80,81,82]. Another study showed that HF diet differentially affected the expression of DNMT1, DNMT3a, and DNMT3b, as well as their binding to the DNA [83]. After eight weeks of post-weaning HF diet intake, DNMT1 binding at the leptin receptor promoter significantly decreased, and DNMT3b binding significantly increased. The study also investigated methyl binding domain protein 2 (MBD2), which may play a role in DNA demethylation. In addition to DNA methyltransferases, it is possible that HF diet differentially affects mediators of active DNA demethylation. In the future, next generation sequencing will allow for a genome-wide view of epigenetic modifier binding in response to dietary challenges.

We provide novel insight into epigenetic programming by HF diet; however, the current study has limitations that should be addressed in future investigation. First, we examine only male offspring in our study. Previously, we have found sex-specific physiological and molecular changes resulting from HF diet intake [84,85,86]. In particular, we have observed large differences in gene expression and DNA methylation in male rats [19,87,88]. In the current study, we build upon prior results in male offspring; however, it is unknown whether these results would be broadly applicable to females. Thus, subsequent studies should examine whether DNA methylation patterns are robust across sexes. Furthermore, we found molecular changes, but we do not provide evidence that these changes induce functional consequences. As this was a genome-wide exploratory study, it was unclear whether certain pathways would be more enriched for changes in DNA methylation. However, now that PI and insulin signaling have been identified, follow-up studies should measure insulin sensitivity and glucose tolerance. Finally, we measured DNA methylation and gene expression at one time point at post-natal week 12, but it is unknown whether DNA methylation is labile between birth, weaning, and further into the post-natal period. It could be the case that younger animals are better equipped than older animals to combat disturbances in the methylome to normalize gene expression. Further work should explore molecular changes at multiple time points to discern whether epigenetic alterations have a similar impact at different life stages.

## 4. Materials and Methods 

### 4.1. Animals and Diets

Timed-pregnant Sprague Dawley rat dams (Charles River Laboratories) were divided into two groups. One group of dams was fed a standard AIN93G diet (*n* = 12; Research Diets, Inc.; 16%, 64%, 20% calories from fat, carbohydrate, and protein, respectively) while the other group was fed a HF diet (*n* = 12; Research Diets, Inc.; 45%, 35%, 20% calories from fat, carbohydrate, and protein, respectively) during gestation and lactation [85]. On post-natal day 21, male pups from control diet-fed dams were weaned onto control diet (*n* = 10) or HF diet (*n* = 10). Male pups from HF-fed dams were weaned onto the AIN93G control diet (*n* = 10). This created three groups of pups: control (CON), post-weaning high fat (PHF), and maternal high fat (MHF). Diet composition is detailed in Table S1. 

Rats were individually housed in standard polycarbonate cages in a humidity- and temperature-controlled room on a 12-hour light-dark cycle with *ad libitum* access to food and drinking water. Body weight and food intake was measured weekly. Each treatment group was kept on their respective post-weaning diet until 12 weeks of age. Before sacrifice, animals were fasted for 12 h and received free access to water. Euthanasia was performed via CO_2_ followed by decapitation. The median lobe of the liver was immediately frozen in liquid nitrogen and stored at −70 °C. Institutional and governmental regulations regarding the ethical use of animals were followed during the study. The protocol for ethical use of animals for this study was approved by the University of Illinois Institutional Animal Care and Use Committee (IACUC protocol no. 09112).

### 4.2. Histological Analysis

Frozen liver samples were embedded in Tissue-Tek OCT compound (VWR, cat. #25608-930) and sectioned, stained, imaged, and quantified using a previously published protocol [86]. Briefly, OCT-embedded liver tissues were sectioned to 7 µm and stained with hematoxylin and eosin (H&E) and Oil Red O solution (Newcomer Supply, cat. #1277A). The slides were imaged using the Nanozoomer imaging system at the Carl R. Woese Institute for Genomic Biology core facilities at the University of Illinois (Hamamatsu Photonics, Hamamatsu City, Japan). Quantification of lipid accumulation was normalized to total protein as previously described [87].

### 4.3. Genomic DNA Isolation

Ten mg of liver tissue was ground in liquid nitrogen and genomic DNA was extracted in 600 μL of Extraction Buffer (50 mM Tris, pH 8.0, 1 mM EDTA, pH 8.0, 0.5% SDS, 1 mg/mL Proteinase K) at 55 °C overnight. Lysate were centrifuged and supernatant was collected and mixed with phenol/chloroform. The mixture was transferred to a phase lock gel tube (Fisher Scientific, cat. #NC1092951) and centrifuged at 16,000× *g* for 5 min for phase separation. The upper phase was transferred and incubated with 1 μL of RNase (Roche, 10 mg/mL) for 1 h at 37 °C. The extraction was then repeated, and the resulting purified DNA was precipitated with 1/10 vol of 3 M Na Acetate (pH 5.2) and 2.5 vol of 100% ethanol. The DNA pellet was washed with 70% ethanol and resuspended in TE. DNA electrophoresis was used to confirm the integrity of the extracted genomic DNA.

### 4.4. MeDIP-Seq and MRE-seq Sequencing

High-throughput sequencing was conducted using liver genomic DNA from each group by complementary MeDIP and MRE methods to measure methylated and unmethylated DNA, respectively. Animals were chosen through an extensive screening process in which gene expression and histology were measured and the best representatives from each group were used for sequencing. Moreover, additional precautions were taken to avoid false positives by using two complementary detection techniques (MeDIP-seq and MRE-seq), setting stringent statistical criteria (details follow), and validating in multiple animals using a probe-based method (details follow).

MeDIP-seq and MRE-seq were conducted in the laboratory of Ting Wang at the University of Washington (St. Louis, MO, USA) using previously established protocols [89]. Briefly, the MeDIP-seq method requires sonication of the DNA into ~500 bp fragments followed by immunoprecipitation using a 5mC-specific antibody. High-throughput sequencing is then used to quantify enriched methylated DNA sequences. MRE-seq method uses restriction enzymes that are sensitive to CpG methylation to cut and enrich unmethylated DNA fragments, thus providing additional coverage and resolution. For MRE-seq, five restriction enzymes were used which have the following recognition sequences CCGG, CCGC, GCGC, ACGT, and CGCG. 

### 4.5. Probe-Based Analysis for Validation of DNA Methylation

A previously established, probe-based method, Quantitative Analysis of Methylated Alleles (QAMA), was modified and used to validate methylation measured by MeDIP-seq and MRE-seq [90]. The method uses two different fluorescently labeled probes, one for methylated DNA and the other for unmethylated DNA, to detect methylated versus unmethylated region of DNA. Genomic DNA was isolated from 16 individual animals using the DNeasy Tissue Kit (Zymo Research, Irvine, CA, USA) and treated with sodium bisulfite reagent using the EZ Methylation Gold kit (Zymo Research). After bisulfite conversion, the DNA sample was diluted to 10 ng/μL for quantitative PCR analysis. The PCR was performed in a 96-well optical plate with a final reaction volume of 20 μL. Each reaction contained 10 μL of Taqman® Universal PCR Master Mix without AmpErase®, 4 μL of nuclease-free water, 1.2 μL of 5 μM each of the forward and reverse primers, and 0.8 μL of 2.5 μM each of the fluorescently labeled methylated and unmethylated probes. The reaction was as follows: 95 °C for 15 min to activate AmpliTaq Gold DNA polymerase followed by 45 cycles of 95 °C for 15 s and 60 °C for 1 min in StepOnePlus real-time PCR system (Applied Biosystems, Foster City, CA, USA). Primer and probe were designed using Primer Express Software 3.0.1 (Applied Biosystems; Table 2). 

Forward and reverse primers were designed to amplify the bisulfite converted DMR sequence. Primers had a melting temperature (Tm) of 58–60 °C and were associated with regions free of CpGs and nucleotide polymorphisms. Two different probes were selected, each with a Tm of 68–70 °C. The methylated probe binds to a sequence with protected (methylated) CpGs while the unmethylated probe binds to a sequence with unprotected (bisulfite converted) CpGs. The methylated probe was labeled with VIC at 5′ and the unmethylated probe was labeled with FAM at 5′, two fluorophores that have absorption/emission spectrums of 538/554 nm and 494/518 nm, respectively. Both probes were labeled with an MGB quencher at the 3′-end. The relative amount of VIC and FAM fluorescence in each reaction was measured and quantities of methylated and unmethylated DNA were analyzed based on VIC and FAM standard curves, respectively. Percentage of methylated DNA was calculated with the following equation:

(quantity of methylated DNA)/(quantity of methylated DNA + quantity of unmethylated DNA)(1)

### 4.6. Methylation Specific PCR

In addition to the probe-based method discussed above, methylation specific PCR (MSP) was used to validate sequencing results. Protocols from previous experimentation were used for primer design, genomic DNA isolation, bisulfite conversion, and qPCR [19]. The percentage of methylated DNA was calculated as the ratio discussed above. All MSP primer information can be found in Table 3.

### 4.7. Gene Expression Analysis

Fifty mg of the liver tissue was homogenized and prepared for mRNA isolation using TRIzol^®^ Reagent (Life Technologies, Carlsbad, CA, USA). Direct-zol™ RNA MiniPrep columns (Zymo Research) with in-column DNase I digestion were used to extract total RNA. RNA concentration and purity were measured using Nanodrop 2000 (Thermo Fisher Scientific, Waltham, MA, USA). Reverse transcription was performed using the High Capacity cDNA Reverse Transcription Kit (Applied Biosystems). The mRNA mixed with reverse transcriptase was incubated in the 2720 Thermal Cycler (Applied Biosystems) at 25 °C for 10 min, 37 °C for 2 h and 85 °C for 5 min. A serially diluted standard curve and all cDNA samples were amplified using Power SYBR® Green Master Mix (Life Technologies) in a StepOnePlus™ Real-Time PCR System (Life Technologies) at 95 °C for 10 min, followed by 35 cycles of 95 °C for 15 s and 60°C for 1 min. A melting curve was included to check the purity of the PCR product. Primers used for quantitative PCR are shown in Table S2. All quantities reported are normalized to β-actin, as it has been shown as a reliable internal control gene and has been used extensively in the past [84,91,92].

### 4.8. DMR Identification

Given the resolution of MeDIP-seq, we partitioned the genome into bins of 500 bp in size and ran MethylMnM to test for statistically different methylation status within each bin (FDR *q*-value < 0.05). The software also outputs MeDIP and MRE RPKM for each animal which was used in subsequent data visualization and analysis. 

### 4.9. Genomic Location of DMRs

DMR location within the genomic context was examined to identify DMR location relative to CpG islands and genes. CpG islands satisfied three criteria: (i) sequences were of length 200 bp or longer, (ii) had guanine-cytosine (GC) content of 50% or greater, and (iii) had a ratio of observed to expected CpGs of 0.6 or higher. Shores were defined as the 2000 bp regions flanking either side of an island [20]. Differentially methylated genes (DMGs) were classified as intragenic, promoter, or downstream regions, where promoter and downstream regions were defined as the 1500 bp sequence upstream of the TSS and the 1500 bp region downstream of the transcription end site, respectively [88].

### 4.10. Pathway Analysis and Functional Clustering

DMGs were annotated using Gene Ontology (GO) terms (Biological Processes (BP), Cellular Component (CC), and Molecular Function (MF)) as well as KEGG pathway enrichment. Functional annotation of DMGs was then used to compute Pearson correlation coefficients (PCC) for pairs of genes to reflect functional similarity between genes. Analysis was performed with the Guide for Association Index for Networks (GAIN) tool available online through the University of Minnesota (http://franklin-umh.cs.umn.edu/similarity_index/index.php) [93]. Given a bipartite network of independent sets, X (genes) and Y (annotation terms), in which interactions are either present or absent, GAIN calculates PCC by considering the likelihood of observing overlap between two vertices in set X given their vertex degree and the total number of Y vertices in the network. A PCC value of 1 represents a perfect functional similarity between two genes, whereas a PCC of 0 indicates no functional similarity. 

Further pathway analysis of DMGs was conducted using DAVID Bioinformatics Resources 6.7 (http://david.abcc.ncifcrf.gov/ [94]). Clusters were formed based on the same GO categories and KEGG pathway enrichments used in the PCC calculations. DAVID analysis generated a hierarchical organization in which related genes were categorized into annotation terms and annotation terms were categorized into annotation clusters. Functional annotation clustering was performed using the following criteria: Similarity Threshold = 0.85 (the minimum similarity value to be considered biological significant), Similarity Term Overlap = 3 (the minimum number of annotation terms overlapped between two genes), Count Threshold = 3 (the minimum number of genes to constitute an annotation term), Minimum Annotation Terms = 3 (the minimum number of annotation terms to constitute an annotation cluster), modified Fisher’s exact *p*-value < 0.01 (lower value indicates higher gene-enrichment within an annotation term), and Enrichment Score > 2.0 (based on the geometric mean of annotation term *p*-values within an annotation cluster). 

### 4.11. General Statistical Analysis

Body weight and food intake were analyzed using ANOVA with repeated-measures followed by post-hoc Tukey HSD test. All gene expression and methylation validation qPCR data were analyzed with R Statistical Software version 3.1.2. Methylation validation was analyzed using a two-tailed student’s *t*-test. Gene expression was analyzed via one-way ANOVA followed by Tukey HSD test.

## 5. Conclusions

To our knowledge, this is the first study to show widespread epigenetic changes in the insulin and PI signaling pathways in response to HF diet. Moreover, we demonstrated that HF diet exposure during either the perinatal or post-weaning period is crucial in determining hepatic DNA methylation profile. Given our results, we predict that perinatal HF diet alters maternal physiology which changes de novo epigenetic modifications in the fetus that persist into adulthood. Post-weaning HF diet, on the other hand, likely works through a more direct mechanism in which HF diet alters methylation maintenance in the mature liver. These distinct processes ultimately result in unique hepatic methylation patterns in the PI and insulin signaling pathways. We also illustrated that genomic location of DMRs, namely their position relative to CpG islands and TSSs, was indicative of gene expression. Our data suggest that maternal and post-weaning HF exposure differentially affect the epigenome within specific genomic contexts.

## Figures and Tables

**Table 1:** QAMA Primers and Probes.

Gene	Position	Sequence
Pklr (+ chr2)	Forward Primer + 4333	5′-TGGTGTTATTTAGATGTTGGAGAGTATGA-3′
	Reverse Primer + 4557	5′-AACATAATACAATCAACCCCATCCA-3′
	Methylated Probe + 4467	5′-VIC-AGGTTCGATTAATTCGGGCG-MGB-3′
	Unmethylated Probe + 4470	5′-FAM-TGATTAATTTGGGTGGAGATAA-MGB-3′
IL-6	Forward Primer − 2516	GTGAGTAAGGGATTTAGTTTGAGTATGGT
	Reverse Primer − 2441	CTTATTCCTAAATATCTAATACCCTCTTATAACCTC
	Methylated Probe − 2480	5′-VIC-CGTGTGTGAATGTGCGTTA-3′
	Unmethylated Probe − 2483	5′-FAM-TTTGTGTGTGAATGTGTGTT-MGB-3′

**Table 2:** Pathway Analysis.

Pathway	DMGs	Fold Enrichment	*p*-Value
Phosphatidylinositol signaling system	11	5.2	0.00004
Insulin signaling pathway	11	2.8	0.006
Axon guidance	10	2.6	0.01
Pathways in cancer	18	1.9	0.01
Purine metabolism	10	2.1	0.04
Glycerolipid metabolism	5	3.8	0.04
Fc gamma R-mediated phagocytosis	7	2.6	0.05

Maternal versus post-weaning HF diet exposure results in seven differentially methylated pathways. DMGs column refers to the number of differentially methylated genes in the pathway. Fold Enrichment signifies the degree of enrichment within each pathway given the overall list of DMGs. A lower *p*-value indicates higher gene-enrichment within a pathway.

**Table 3:** MSP Primers.

Gene	Position	Sequence
*Acacb*	Forward M + 75,096	5′-TTGGGTTCGGTTTTTAGTTTCG-3′
	Reverse M + 75,222	5′-ACGTATATCCCTATAATCCAACTCGC-3′
	Forward UM + 75,095	5′-TTTGGGTTTGGTTTTTAGTTTTGAA-3′
	Reverse UM + 75,225	5′-CACATATATCCCTATAATCCAACTCACTCT -3′
*Gpam*	Forward M + 7289	5′-AGTCGTAGTGGTCGGGTAATCG-3′
	Reverse M + 7357	5′-CCGCTTATTTTAAACAACATCGAA-3′
	Forward UM + 7288	5′-AAGTTAAGTTGTAGTGGTTGGGTAATTG-3′
	Reverse UM + 7360	5′-CCCACTTATTTTAAACAACATCAAACC-3′

## Supplementary Materials

Supplementary materials can be found at https://www.mdpi.com/1422-0067/20/13/3229/s1.

## Abbreviations

HF	high-fat diet
MeDIP-seq	methylated DNA immunoprecipitation with high-throughput sequencing
MRE-seq	methylation-sensitive restriction enzyme digestion sequencing
DMR	differentially methylation regions
DMG	differentially methylated genes
Mc4r	melanocortin 4 receptor
Ppara	peroxisome proliferator-activated receptor α
*Tlr-1* and *Tlr-2*	toll-like receptors 1 and 2
*Cdkn1a*	cyclin-dependent kinase inhibitor
*H&E*	hematoxylin and eosin
*QAMA*	Quantitative Analysis of Methylated Alleles
*MSP*	methylation specific PCR
*GO*	Gene Ontology
*KEGG*	Kyoto Encyclopedia of Genes and Genomes
*PCC*	Pearson correlation coefficients
