# Supplementary material for: Maternal and Post-Weaning High-Fat Diets Produce Distinct DNA Methylation Patterns in Hepatic Metabolic Pathways within Specific Genomic Contexts
Source: Int J Mol Sci. 2019 Jun 30;20(13):3229. doi: 10.3390/ijms20133229 (PMC6651091; doi:10.3390/ijms20133229)
Supplement: Supplementary file 1 [file ijms-20-03229-s001.pdf]

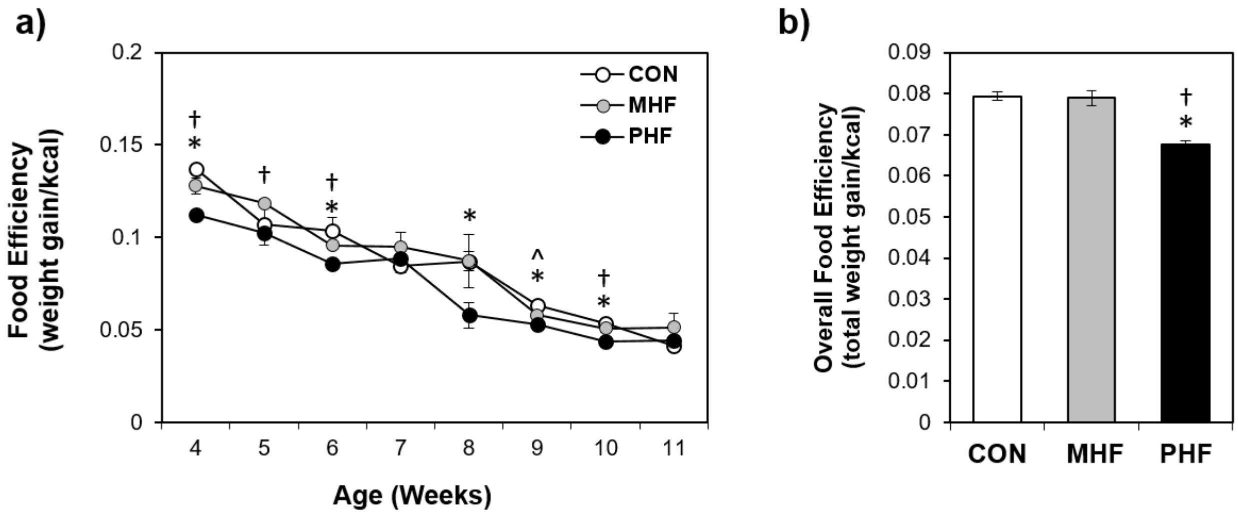

Fig S1. Food efficiency. a) Food intake (kcal) and body weight gain (grams) were measured weekly during the post-weaning period. b) Total weight gain over the duration of the post-weaning period was divided by the total energy intake during the same timeframe. *denotes a significant difference ($p<0.05$) between PHF and CON. † denotes a significant difference between PHF and MHF. ^ denotes a significant difference between MHF and CON.

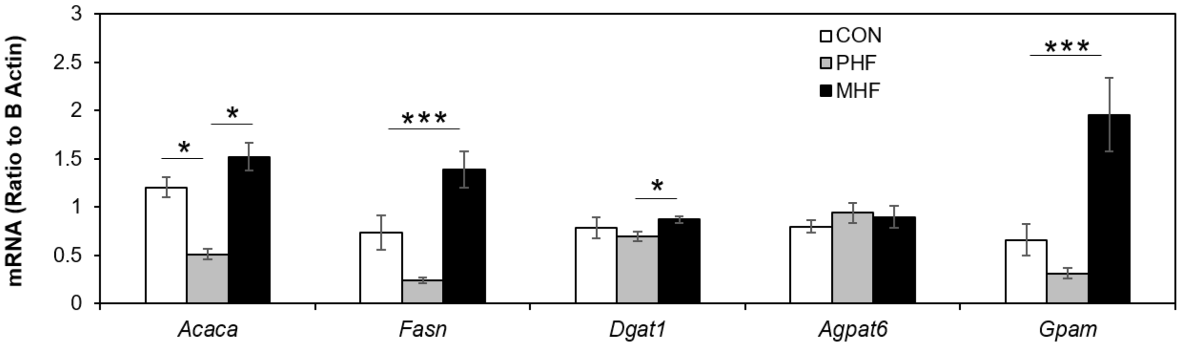

Fig S2. Expression of genes related to de novo lipogenesis and triglyceride synthesis. qPCR was used to measure hepatic gene expression. *Acaca*: acetyl-CoA carboxylase alpha, *Fasn*: fatty acid synthase, *Dgat1*: diacylglycerol O-acyltransferase 1, *Agpat6*: glycerol-3-phosphate acyltransferase 4, *Gpam*: glycerol-3-phosphate acyltransferase (mitochondrial). mRNA quantity is normalized to B Actin. Data are presented as mean ±SEM. *denotes a significant difference ($p<0.05$) between the designated groups. ***denotes a significant difference between all three groups.

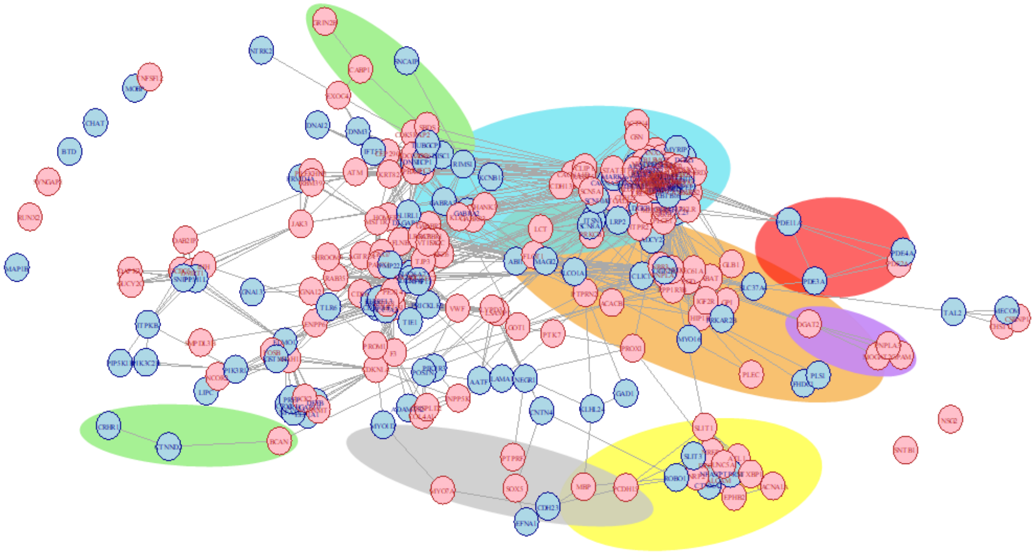

Cluster 1	ion binding	Enrichment Score: 5.9
Cluster 2	cell morphogenesis	Enrichment Score: 5.1
Cluster 3	cell fraction	Enrichment Score: 4.9
Cluster 4	cell morphogenesis involved in differentiation	Enrichment Score: 4.0
Cluster 5	stereocilium	Enrichment Score: 3.1
Cluster 6	regulation of synaptic transmission	Enrichment Score: 3.0
Cluster 7	triglyceride biosynthetic process	Enrichment Score: 2.4
Cluster 8	cAMP catabolic process	Enrichment Score: 2.3

Fig S3. Functional similarity between DMGs based on Gene Ontology and KEGG Pathway analysis. Gene Ontology annotations (Biological Process, Molecular Function, and Cellular Component) and KEGG pathways (DAVID) were used to annotate DMGs. There are a total of 8 distinct clusters identified (Enrichment score > 2.0, p < 0.01). Blue vertices indicate DMGs that are more methylated in the MHF group. Red vertices indicate DMGs that are more methylated in the PHF group. Edges represent Pearson correlation coefficient greater than 0.5.

Ingredient	Control (AIN-93G) g/1000 kcal	Control (AIN-93G) kcal	HF g/1000 kcal	HF kcal
Casein	49.8	199.2	49.8	199.2
L-Cystine	0.7	3	0.7	3
Cornstarch	108.8	435.4	18.1	72.5
Maltodextrin	24.9	99.6	24.9	99.6
Sucrose	25.4	101.6	43.0	172
Cellulose	12.4	0	12.4	0
Soybean Oil	6.2	56	6.2	56
−Saturated	1	9	1	9
−Monounsaturated	1.5	13.4	1.5	13.4
−Polyunsaturated	3.7	33.6	3.7	33.6
Lard	11.7	105.3	44.2	397.7
−Saturated	4.9	44.2	18.6	167
−Monounsaturated	5.3	47.4	19.9	179
−Polyunsaturated	1.5	13.7	5.7	51.7
Total saturated fat	5.9	53.2	19.6	176
Total monounsaturated fat	6.8	60.8	21.4	192.4
Total polyunsaturated fat	5.3	47.3	9.5	85.3
Mineral mix[1]	2.5	0	2.5	0
Dicalcium phosphate	3.2	0	3.2	0
Calcium carbonate	1.4	0	1.4	0
Potassium citrate	4.1	0	4.1	0
Vitamin mix[2]	2.5	0	2.5	0
Choline bitartrate	0.5	0	0.5	0

Table S1. Diet composition. Both HF and control diets were purchased from Research Diets Inc. (New Brunswick, NJ, USA). [1]Mineral mix (AIN-93): Research Diets product no. S10026 for rodents. [2]Vitamin mix (AIN-93): Research Diets product no. V10001 for rodents.

Table S2. List of primers used for qPCR.

Gene Name	Ensembl ID	Position	Sequence	Amplification Efficiency*
Acacb	ENSRNOE00000005256	Forward +1081	5'-ACCCCAAACTTCCAGAGC-3'	114.62%
		Reverse +1189	5'-TGGGCTACAATGGTGGAG-3'	
Akt	ENSRNOT00000025303	Forward +1545	5'-CTCCTGCCACCCTTCAAAC-3'	108.15%
		Reverse +1624	5'-ATGGACTGGGCGGTGAAT-3'	
Apkc	ENSRNOT00000021285	Forward +406	5'-AGGGACGAAGTGCTCATCAT-3'	96.53%
		Reverse +479	5'-TCTTCTCCAGGACAAGGCAT-3'	
B Actin	ENSRNOT00000042459	Forward +451	5'-GAGACCTTCAACACCCCAGC-3'	96.26%
		Reverse +526	5'-CAGTGGTACGACCAGAGGCA-3'	
Cbl	ENSRNOT00000002719	Forward +1955	5'-TATGAGGAAACACAGACGCCACGA-3'	94.81%
		Reverse +2034	5'-ATTCTTCAGGGGCAAGGTGTCCAT-3'	
Dgkg	ENSRNOT00000002452	Forward +272	5'-CATCCGTGGGTCTTGAAGCGA-3'	106.10%
		Reverse +335	5'-AGACAGACCCATTGCCCGTCA-3'	
Dgkb	ENSRNOT00000063874	Forward +981	5'-GGGCTTGGAAAACAATGTGAAGG-3'	104.21%
		Reverse +1052	5'-TAGGCGGGCTTGTTAAAGTGCTT-3'	
Flotillin	ENSRNOT00000001098	Forward +324	5'-AGAGTGAGAAGGTTTATACCCGCCA-3'	99.13%
		Reverse +403	5'GTTTTGCCCCTGGATTTTCACCT-3'	
Glut4	ENSRNOT00000027910	Forward +647	5'-GGGTGCCTTGGGAACACTCAAC-3'	93.43%
		Reverse +710	5'-CAACACCTGGGCAACCAGAATG-3'	
Hsl	ENSRNOT00000027910	Forward +2697	5'-GCAAGCCCCATAAGACCCCA-3'	107.23%
		Reverse +2775	5'-CGCATAGACTCCGTAAGTCGCC-3'	
Inpp5k	ENSRNOT00000048371	Forward +356	5'-GCTTTCTCCACTGAACCTTGTC-3'	100.58%
		Reverse +425	5'-GGCAAAGACCAGTAAGAGAAGC-3'	
Insr	ENSRNOT00000067448	Forward +2200	5'-CAAACAGATGCCACCAATC-3'	103.85%
		Reverse +2265	5'-GAGATGAGGAGTTGGAAACTG-3'	
Ip3k	ENSRNOT00000004032	Forward +3038	5'-AGAAACCCTGGAAGTCTC-3'	102.34%
		Reverse +3113	5'-CTTGTCGTGGATGAAGAG-3'	
Irs	ENSRNOT00000019579	Forward +307	5'-AGGACTGGGGGAGACTTAGTC-3'	101.18%
		Reverse +384	5'-TCATGCCCAGAGGGAAAG-3'	
Lar	ENSRNOT00000027271	Forward +1661	5'-CTGGGCAGCAGAGGATGAAGG-3'	96.37%
		Reverse +1727	5'-CAGAGTGTAGGAGGAGGTGGGGTC-3'	
Pde3	ENSRNOT00000032843	Forward +1199	5'-AGAACTATACCTGCTCCGAC-3'	99.50%
		Reverse +1278	5'-CAAACTTCTTCTCAGGCG-3'	
PI Synthase	ENSRNOT00000013691	Forward +839	5'-GAACTCTTCTACTGCCTCCTGTACC-3'	110.73%
		Reverse +915	5'-ATTCGGAAAAGCCCCACAG-3'	
Pik3c2b	ENSRNOT00000042125	Forward +1730	5'-ATGTGGGCGACTTTGTGCTCAA-3'	90.05%
		Reverse +1797	5'-TCCCAAGGCGTGCTTATTTTGC-3'	
Pi4k	ENSRNOT00000065378	Forward +293	5'-TCGGAAAGGCAGAGGTAA-3'	110.38%
		Reverse +365	5'-CACATCAGACAGCAAGGTCA-3'	
Pik3r1	ENSRNOT00000025687	Forward +879	5'-AATCTCAACGGAGTGGAG-3'	106.57%
		Reverse +951	5'-TATAGAAGTGGGCTTGGG-3'	
Pik3r3	ENSRNOT00000000157	Forward +884	5'-TGAATAGCATCAAGCCTGACC-3'	100.92%
		Reverse +955	5'-TGTGATTGAGCCATACGAGGT-3'	
Pipk	ENSRNOT00000068645	Forward +770	5'-CAGGTTTAAGACATACGCC-3'	112.33%
		Reverse +849	5'-AGTACAAGTAATCGTCGGG-3'	
Phk	ENSRNOT00000065678	Forward +1556	5'-CTTTTACACCCCAGTTCACTGACCA-3'	97.08%
		Reverse +1631	5'-CTCAGCATCTCCACAATCATCTCGT-3'	
Pka	ENSRNOT00000012415	Forward +370	5'-GCTACAATCACTGCTACCTCTC-3'	92.64%
		Reverse +435	5'-TATTCTCCTGAAGGTCACCC-3'	
Plc	ENSRNOT00000067149	Forward +1994	5'-AAGCAGCCTTCTCAGTTCCTTTGG-3'	111.55%
		Reverse +2057	5'-CGGTTGGCGTTCTTTCAGCATT-3'	

Pp1	ENSRNOT00000051720	Forward +838	5'-TATGAAAGAATGGAGTTCGCCGTG-3'	98.56%
		Reverse +909	5'-TTTGCCTTTGTTGCTGTCCCAGTA-3'	
Pygl	ENSRNOT00000009183	Forward +1019	5'-CTCCAAGGATGGTGTAGGAA-3'	102.61%
		Reverse +1097	5'-TGCGGGATGTGTGTCATT-3'	
Pklr	ENSRNOT00000027700	Forward +655	5'-ATTGACGACGGGCTCATCTCCT-3'	100.25%
		Reverse +734	5'-ATACCACCGTGCTCCACTTCTGTC-3'	

*The qPCR amplification efficiency is calculated based on the slope of the standard curve https://www.lifetechnologies.com/us/en/home/brands/thermo-scientific/molecular-biology/molecular-biology-learning-center/molecular-biology-resource-library/thermo-scientific-web-tools/qpcr-efficiency-calculator.html. Slopes between -3.1 and -3.6 giving reaction efficiencies between 90 and 110% are typically acceptable.
